# The Effect of Propolis As A Biological Storage Media on Periodontal Ligament Cell Survival in An Avulsed Tooth: An *In Vitro* Study

**Published:** 2013-08-24

**Authors:** Zohreh Ahangari, Samiye Alborzi, Zahra Yadegari, Fatemeh Dehghani, Leila Ahangari, Mandana Naseri

**Affiliations:** 1 Department of Endodontics, Dental School, Shahid Beheshti University of Medical Sciences, Tehran, Iran; 2 Student Research Committee, Shahid Beheshti University of Medical Sciences, Tehran, Iran; 3 Oral Biology Laboratory, Dental School, Shahid Beheshti University of Medical Sciences, Tehran, Iran; 4 Islamic Azad University, North Tehran Branch,Tehran, Iran

**Keywords:** Avulsed Tooth, Periodontium, Propolis, Transport Media

## Abstract

**Objective:**

Both the length of extra-alveolar time and type of storage media are significant factors that can affect the long-term prognosis of replanted teeth. This study aims to compare propolis 50%, propolis 10%, Hank’s balanced salt solution (HBSS), milk and egg white on periodontal ligament (PDL) cell survival for different time points.

**Materials and Methods:**

: In this* in vitro* experimental study, we divided 60 extracted teeth without any periodontal diseases into five experimental and two control groups that consisted each experimental group with 10 and each control group with 5 teeth. The storage times were one and three hours for each media. The controls corresponded to 0-minute (positive) and 12-hour (negative) dry time. Rinsing in the experimental media, the teeth were treated with dispase and collagenase for one hour. Cell viability was determined by using trypan blue exclusion. Statistical analysis of the data was accomplished by using two-way analysis of variance (ANOVA) complemented by the Tukey’s HSD post-hoc.

**Results:**

Within one hour, there was no significant difference between the two propolis groups, however these two groups had significantly more viable PDL cells compared to the other experimental media (p<0.05). The results of the three-hour group showed that propolis 10% was significantly better than egg white, whereas both propolis 10% and 50% were significantly better than milk (p<0.05).

**Conclusion:**

Based on PDL cell viability, propolis could be recommended as a suitable biological storage media for avulsed teeth.

## Introduction

ulsion is defined as the total displacement of a tooth from the alveolar socket. Clinical surveys indicate that avulsion occurs in 1to 16% of all traumatic injuries to permanent dentition (1). Extra oral time of the avulsed tooth and storage medium are two important factors that prevent damage to the periodontal ligament cells (PDL) (2). Immediate replantation of the tooth preserves the vitality of the PDL cells, which is an essential factor for increasing long term prognosis (3).Water is more acceptable than desiccation as storage for up to 15 minutes. Milk is acceptable for up to 60 minutes; cool milk reduce swelling and increases cell viability (3). Hank’s balanced salt solution (HBSS) is non-toxic, pH- balanced and contains many essential nutrients. Although HBSS is available as "Save-A- Tooth", a standard storage media, it is not widely used (4, 5).

Recently propolis has been recognized as a useful material for human and veterinary medicine. It is an antifungal (6), antibacterial and anti-inflammatory resinous material collected by bees from the buds of plants (7). In general, propolis is composed of 50% resin and vegetable balsam, 30% wax, 10% essential and aromatic oils, 5% pollen and 5% various other substances, that include organic debris depending on the place and time of its collection (8, 9). The constituents of propolis vary widely due to climate, season and location, therefore its chemical formula is not stable (10, 11).

The effect of this media on PDL cells was examined in several studies and it has been reported to be more effective than HBSS (12, 13). In one study there was no statistical difference in the viability of PDL cells between egg white and HBSS as storage media, but both were more effective than milk (14).

In this study, we compared the viability of PDL cells using milk and egg white as available storage media, HBSS as the golden standard storage media approved by the American Association of Endodontists (2, 3, 15), and two different concentrations of Iranian propolis as a new biological storage media for the maintenance of viable PDL cells.

## Materials and Methods

This was an* in vitro* experimental study that examined a total of 60 periodontal disease-free anterior single root teeth which, due to prosthodontic consideration, were extracted without trauma. The teeth were randomly divided into five experimental and two control groups five each (Save-A-Tooth, Pottstown, PA, USA). After drying for 30 minutes, a total of 10 teeth were used for each of the following experimental media: propolis 10%, propolis 50%, Hank’s balanced salt solution [HBBS (Save-A-Tooth, Pottstown, PA, USA)], milk and egg white. We evaluated each of the groups at one and three hours after immersion, with a total of five teeth used for each immersion.

**Fig 1:**
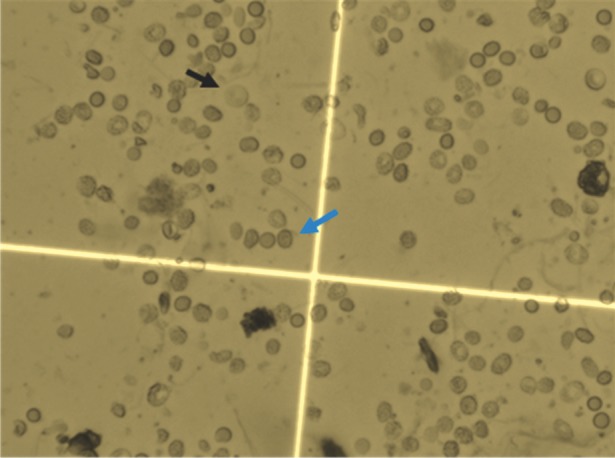
non-viable (blue arrow) periodontal cells labeled by trypan blue under light microscope (×400).

Propolis was produced by honeybees in Azerbaijan, Iran. Solid propolis was ground by a mortar and pestle, then divided into 1.6 and 8 mg portions and dissolved in 40 ml of the 0.4% ethanol solution by a using vortex mixer (Techno Inc., USA) to make propolis 10 and 50%, respectively. The propolis extract was filtered to exclude any rough particles. Prior to immersion of the teeth in prepared propolis, the solutions were agitated well by a shaker. After drying and soaking in all storage media for the determined time, each tooth was incubated in 15 ml falcon tubes with 0.8 ml of collagenase (1.3 mg/ml) (Gibco, UK) and also 0.8 ml dispase (0.5 mg/ml) (Gibco, UK) for one hour in a water bath at 37˚C. The tubes were shaken every ten minutes. We added 8 ml of fetal bovine serum (FBS) (Gibco, Australia) to each tube, after which tubes were centrifuged for ten minutes at 1200 rpm for cell separation. A total of 50 ml of 0.4% trypan blue (Merck, Germany) was added to label the cells for determination of cell viability. Cell viability was calculated as the number of viable cells divided by the total number of cells within the grids on the hemocytometer at ×400 magnification under a light microscope. Cells that took up trypan blue were considered non- viable (Fig 1). We calculated the viable cell count as follows:

% viable cells=[1.00-(Number of blue cells/ Number of total cells)]×100

We treated the control groups with dispase and collagenase. The positive control group were treated immediately after extraction whereas the negative ones were treated after leaving to dry for 12 hours. All the procedures were done by a skilled practitioner who was blinded to the treatment.

### Statistical analysis

Statistical analyses were conducted using a twoway analysis of variance (ANOVA) complemented by the Tukey’s HSD post-hoc test. The level of significance was p<0.5. The analyses were performed using SPSS17.

## Results

The mean percent values for cell viability for the different groups are shown in table 1. There was no significant difference in the one-hour groups between HBSS (62.97 ± 8.44), milk (63.82 ± 4.68) and egg white (59.79 ± 5.54) in numbers of remaining vital cells (p>0.05). Although there was no significant difference between the two propolis groups, both propolis 50% (77.78 ± 9.22) and propolis 10% (78.55 ± 3.64) were significantly better than the other experimental groups (p<0.05).

The milk (34.64 ± 26.80) and egg white (50.38 ± 11.31) three-hour groups were not significantly different. There was no significant difference between propolis 50% (64.00 ± 15.16), propolis 10% (79.97 ± 2.54) and HBSS (60.23 ± 7.78), either (p>0.05). Propolis 10% was significantly better than the egg white group, whereas both propolis 50% and propolis 10% were significantly better than the milk group (p<0.05).

**Table 1:** The mean values and standard deviations of alive to total periodontal ligament (PDL) cells in study groups


Groups	1hour (no/unit)	3 hours (no/unit)

**Egg white**	59.79 ± 5.54	50.38 ± 11.31
**Milk**	63.82 ± 4.68	34.64 ± 26.80
**HBSS**	62.97 ± 8.44	60.23 ± 7.78
**Propolis 50%**	77.78 ± 9.22	64.00 ± 15.16
**Propolis 10%**	78.55 ± 3.64	79.97 ± 2.54
**Positive control**	80.33 ± 24.15	
**Negative control**	3.92 ± 5.70	


HBSS; Hank’s balanced salt solution.

## Discussion

Avulsions are the worst dentoalveolar injuries. Successful tooth replantation is directly dependent on the viability of PDL cells that remain on the root surface (16). Numerous studies have examined the best storage medium for PDL cell viability or the critical dry time that cells remain viable.

In this study, as with the study by Martin and Pillegi, the dry time was considered to be 30 minutes (12). Andreasen and Hjorting-Hansen (17) showed that teeth replanted within 30 minutes had a better chance for survival than longer periods of dry time. In addition, 30 minutes of desiccation is similar to the clinical setting. Other studies have shown that with a two hour dry time no vital PDL cells remain (18, 19). In addition, other investigations have used a dry time that differed from the normal clinical setting (13, 14, 20, 21).

According to previous studies that examined different storage media, the results showed that water, saliva, and saline were ineffective on PDL cell vitality because of bacterial contamination or the hypotonic effect that lead to the death of PDL cells (15).

HBSS has been used in numerous studies to protect different cell types. It is non-toxic, pH balanced, and contains many essential nutrients. HBSS is available as "Save-A-Tooth" in pharmacies, but not widely (4, 5).

Lindskog et al. in an *in vivo* study on monkeys evaluated saliva and milk. The results have shown that saliva was less suitable than milk due to its low osmolality and bacterial contamination (22). Milk, however, is a suitable transport medium because of its pH=6.5-6.8 and osmolality of 275 milliosmol/kg, in addition to the presence of essential nutrients (4).

Viaspan is presently used for organ transplant storage. It’s lactobionate and raffinose are presumed to prevent cellular swelling (23). Viaspan has an effective hydrogen ion buffer, which maintains the pH, in addition to adenosine, which is necessary for cell division (24). Pettiette and colleagues (25) have shown that viaspan was more effective than HBSS and milk, however other studies disagreed (26). Viaspan has a pH=7.4 and osmolality of 320 milliosmol/ kg, which has proven that it is among the most effective of media. However, viaspan is not widely available (4).

Propolis is an aromatic sticky substance collected by bees from trees and plants. The bees work on it to produce a type of adhesive that seals their hives (7). Propolis is composed of resin (55%), essential oils and wax (30%), pollen (5%) and other constituents (10%) that consist of amino acids, minerals ethanol, in addition to vitamins A, B complex, E and bioflavonoid (7).

cterial, anti-viral and antifungal properties. It has been used in dentistry for surgical wound healing (27, 28), root canals (29, 30), periodontal treatments (31, 32), and pulp capping (33), in addition to research as a media storage for avulsed teeth (12, 13).

There are three methods in use for evaluating the efficacy of different storage media. One method initially removes fibroblasts from the root surfaces after which they are added to a storage medium for culturing. The viability of cells is evaluated at different times. The advantage of this method is the large number of cells produced, however this differs from the clinical setting (34). Another method, after tooth extraction and different dry times, soaks the tooth in storage media. By isolating the tooth and using enzymes, the cell viability is evaluated. This method more closely approximates the actual clinical scenario. In the third method, apoptotic levels are determined by an apoptosis assay and flow cytometry.

Additional cell viability and proliferation are analyzed by the XTT assay under both dry and wet conditions. The present study used the second method, as have Martin and Pillegi (12) and Khademi et al. (14), in contrast with research by Ozan et al. (13, 20) and Thomas et al.

(21) who used the first method and Gjertsen et al. (35) who has used the third method. We have used the trypan blue exclusion staining technique because it is quick, easy, and can differentiate between viable and nonviable cells.

The results of present study showed that both propolis 10 and 50% were more effective than other groups at the studied times, however there was no difference between propolis 10 and 50%. After one hour, milk and egg white were observed to be effective as HBSS but significantly less effective than both propolis groups. At three hours, the effects of both concentrations of propolis were the same as HBSS, whereas milk and egg white were less effective than HBSS and both propolis groups.

Martin and Pillegi (12) showed that propolis 100% and propolis 50% were the most effective storage media, with no statistical differences between these two groups, also there was no difference between HBSS, milk and saline. Although the percent of propolis in their study differed from the current study, the results were similar.

Khademi et al. (14) reported no significant difference between HBSS and egg white at any storage time. Both were more suitable than water or milk. In the present study there was no significant difference between milk and egg white at both intervals. The use of different enzymes or lack of dry time in Khademi and his colleagues’ investigation might have led to different results.

Ozan et al. (13) compared the efficacy of propolis 10%, propolis 20%, milk and HBSS. He found that propolis was significantly more effective than HBSS and milk at 3, 6, 24, 72 hours. However at one hour no significant difference existed among the groups. Propolis 20% had a worse result than HBSS but better than HBSS and milk at three and six hours. No difference existed between propolis groups at one, three and six hours. In their study milk kept significantly less numbers of PDL cells viable compared to HBSS, which supported the results of the current study.

Thomas et al. (21) compared coconut water, HBSS and milk on PDL cell survival. He found HBSS to be more effective at 15 minutes. No significant difference existed among groups at 30, 45 and 60 minutes. The results of this study resembled ours in the HBSS and milk groups.

Gopikrishna et al. (36) compared coconut water, propolis 50%, HBSS and milk on PDL cell viability and found coconut water to be the most effective group. Propolis 50% and HBSS were more effective than milk, such as our results.

Buttke and Trope have suggested that storing avulsed teeth in medium that contain antioxidants might increase replantation success (37). One of the major components of propolis is flavonoids, the most important pharmacologically active constituent and powerful antioxidant, which would explain its ability to maintain cell viability. Propolis also has an antibacterial property, which assists with successful replantation and decreases the chance of inflammatory resorption-a common sequel in delayed replantation. Iron and zinc which are used in collagen synthesis are also found in propolis.

## Conclusion

Many storage media can be considered for avulsed teeth according to the circumstances and properties of the natural or chemical cellular protective solutions.

 obtained in the present study, propolis could be suggested as a suitable storage medium for maintaining the viability of PDL cells in avulsed teeth. Further research is needed to produce a standard formulation for propolis in addition to studies on animal models of traumatic injuries.
